# Molecular Characterization of Heat Shock Protein 70-1 Gene of Goat (*Capra hircus*)

**DOI:** 10.4061/2010/108429

**Published:** 2010-06-29

**Authors:** Nitin Gade, R. K. Mahapatra, Arvind Sonawane, V. K. Singh, Ramesh Doreswamy, Mohini Saini

**Affiliations:** ^1^Division of Physiology and Climatology, Indian Veterinary Research Institute, Izatnagar, Uttar Pradesh, 243 122, India; ^2^Division of Animal Genetics, Indian Veterinary Research Institute, Izatnagar-243 122, Uttar Pradesh, India; ^3^Centre for Wildlife, Indian Veterinary Research Institute, Izatnagar-243 122, Uttar Pradesh, India

## Abstract

Heat shock protein 70 (HSP 70) plays a vital role by bestowing cytoprotection against diverse kinds of stresses. The ubiquitous HSP 70 proteins are the most abundant and temperature sensitive among all the HSPs. The present paper has characterized HSP70-1 cDNA in goat (*Capra hircus*). Total RNA isolated from goat peripheral blood mononuclear cells was reverse transcribed to cDNA that was used for amplification of HSP 70-1 gene. PCR product (1926 bp) was cloned in pGEM-T easy vector and sequenced. Sequence analysis revealed 1926-bp-long open reading frame of HSP 70-1 gene encoding 641 amino acids in goat, as reported in cattle. At nucleotide level, goat HSP 70-1 was found to be 96–99% similar to that of sheep (partial), cattle, and buffalo whereas the similarity at amino acid level was 95–100%. Nonsynonymous substitutions exceeding synonymous substitutions indicate the evolution of this protein through positive selection among domestic animals. Goat and sheep appear to have diverged from a common ancestor in phylogenetic analysis. Predicted protein structures of goat HSP 70 protein obtained from deduced amino acid sequence indicated that the functional amino acids involved in chaperoning through ATPase hydrolytic cycle and in uncoating of clathrin coated vesicles are highly conserved.

## 1. Introduction

Ritossa in 1962 [1] first observed cellular stress response in *Drosophila busckii *salivary glands which were exposed to high temperature. Heat shock genes are activated when cells are exposed to stress stimuli and form heat shock proteins [2]. Heat shock proteins family consists of many proteins which are classified as HSP 110, HSP 100, HSP 90, HSP70, HSP 60, HSP 40, HSP 10, and small HSP families [3]. Among HSPs, heat shock protein 70 (HSP70) is an essential molecular chaperone of primary importance to all mammalian cells. HSP70 gene family in bovines includes HSP70-1, HSP70-2, HSP70-3, and HSP70-4 gene. HSP70-1 is an intronless gene located on chromosome 23 of bovine (BTA 23) and has 1926 nucleotides. HSP70 is well reported to protect cells, tissues, and organs from stress [4] by promoting the folding of nascent polypeptides and by correcting the misfolding of denatured proteins. Heat shock-induced-HSP70 expression has a role in the antiapoptotic pathway [5]. 

Goat (*Capra hircus*) is one of the oldest domesticated species which was found in the Indian subcontinent since 9000 B.C.E. [6]. Goat rearing is an important economic and social activity for resource-limited farmers of developing countries like India. Among the domestic ruminant species, goats are less susceptible to environmental stress as they possess water conservation capability, higher sweating rate, lower basal metabolism, higher respiration rate, higher skin temperature, and constant heart rate and cardiac output [7]. But in Indian conditions goats usually left for grazing during day time are prone to heat stress due to high ambient temperature which could adversely affect their productivity and energy efficiency. Restriction fragment length polymorphism (RFLP) analysis in goat revealed the location of HSP70 genes within the major histocompatibility complex class I [8]. Similar to bovines, goats also possess four HSP70 genes out of which HSP70-3 gene sequence in Shiba goat has been characterized by Luengrattana and coworkers [9]. The spontaneous expression of HSP70 protein is mainly due to the transcription of the major gene, HSP70-1 [10]. Scanty reports are available regarding the role of HSP70 during heat stress in goat, and HSP70-1 gene has not been characterized in this species. The characterization of HSP70-1 gene will be helpful for deriving phylogenic relationship among different species and for determining expression and identifying new functions among the related species. Considering the importance of HSP70-1 gene in conferring thermotolerance, present paper reports its characterization in goat and its comparison with that of other farm animals.

## 2. Materials and Methods

### 2.1. Animals, Sample Collection, and RNA Isolation

Blood was collected aseptically by jugular vein puncture over 2.7% ethylenediamine tetraacetic acid from healthy goat (*capra hircus*) reared in animal shed at Physiology and Climatology Division, IVRI. Peripheral blood mononuclear cells (PBM cells) were isolated from whole blood by density gradient centrifugation [11] using Lymphocyte Separation Media (LSM) (Himedia). The total RNA was isolated from separated cells using TRIzol (Life Technologies, USA), following manufacturer's instructions and was further used for cDNA synthesis.

### 2.2. Synthesis and Confirmation of cDNA PCR Amplification of HSP70-1 Gene

The 20 *μ*L reaction mixture contained 5 *μ*g of total RNA, 0.5 *μ*g of oligo dT primer (16–18 mer), 40 U of Ribonuclease inhibitor, 1000 *μ*M of dNTP mix, 10 mM of DTT, and 5 U of MuMLV reverse transcriptase in 5× reverse transcriptase buffer. The reaction mixture was gently mixed and incubated at 37°C for 1 hour. The reaction was stopped by heating the mixture at 70°C for 10 minutes and chilled on ice. The integrity of the cDNA checked by PCR with GAPDH primers which amplified 200-bp-GAPDH gene fragment. To amplify full length open reading frame (ORF) of HSP70-1 gene sequence, a specific primers pair was designed based on the HSP70-1 mRNA sequences of cattle (Accession No- NM_174550) by using Primer select programme of DNA star software. The primers were Forward: HSP70-1-F 5′ATGGCGAAAAACATGGCTATC3′ (21 mer) and Reverse: HSP70-1-R 5′CTAATCCACCTCCTCAAT3′ (18 mer). The 50-*μ*L PCR reaction contained 50 pmol of each forward and reverse primers, 1-*μ*L template cDNA, 200 *μ*M of dNTP mix, 1.0 mM MgCl_2_, and 3 U proofreading DNA polymerase (MBI Fermentas, USA) in 1 × Taq buffer. Amplification conditions were as follows: initial denaturation at 94°C for 2 min, 35 cycles at 94°C for 1 min, annealing at 49°C for 45 sec, and extension at 72°C for 2.20 min, followed by a final extension at 72°C for 10 min.

### 2.3. cDNA Cloning and Sequencing

PCR amplicons verified by 1% agarose gel electrophoresis were purified from gel using Gel extraction kit (Qiagen GmbH, Hilden, Germany) and ligated into pGEM-T easy cloning vector (Promega, Madison, WI, USA) following manufacturers' instructions. The 10 *μ*L of ligated product was directly added to 200 *μ*L competent cells, and heat shock was given at 42°C for 45 sec. in a water bath, and cells were then immediately transferred on chilled ice for 5 min., and SOC was added. The bacterial culture was pelleted and plated on LB agar plate containing Ampicillin (100 mg/mL) added to agar plate @1 : 1000, IPTG (200 mg/mL) and X-Gal (20 mg/mL) for blue-white screening. Plasmid isolation from overnight-grown culture was done by alkaline lysis method [12] with little modification as described by Sambrook and Russell [13]. Recombinant plasmids were characterized by PCR using gene-specific primers and restriction enzyme digestion based on reported nucleotide sequence for cattle. The enzyme Bsr I (MBI Fermentas, USA) was used for restriction analysis of amplified PCR product, EcoR I (MBI Fermentas, USA) for fragment release, whereas Sac I (MBI Fermentas, USA) and Pst I (MBI Fermentas, USA) for insert characterization. HSP70-1 gene fragment insert in recombinant plasmid was sequenced using M13 Forward and Reverse primer pair by primer walking using an automated DNA sequencer (Chromous Biotech, Bangalore). 

### 2.4. Sequence Analysis

The nucleotide sequence so obtained was analyzed for protein translation, sequence alignments, and contigs comparisons by DNASTAR Version 4.0, Inc., USA. Phylogenetic tree based on the evolutionary distances was constructed from nucleotide sequences using MEGA 4 software. Based on the nucleic acid alignment, the number of synonymous substitution per synonymous site (dS) and number of nonsynonymous substitution per nonsynonymous sites (dN) were estimated, and neutral (dS = dN), positive (dN > dS), or purifying (dN < dS) selections were tested with a codon-based *Z* test using Nei Gojobori method. Novel sequence was submitted to the NCBI Genbank and accession number was obtained which is available in public domain now. Goat HSP70 protein structure was predicted by the Simple Modular Architecture Research Tool (SMART)(http://smart.embl-heidelberg.de/).

## 3. Results and Discussion

### 3.1. Characterization of HSP70-1 Gene

**Figure 1:**
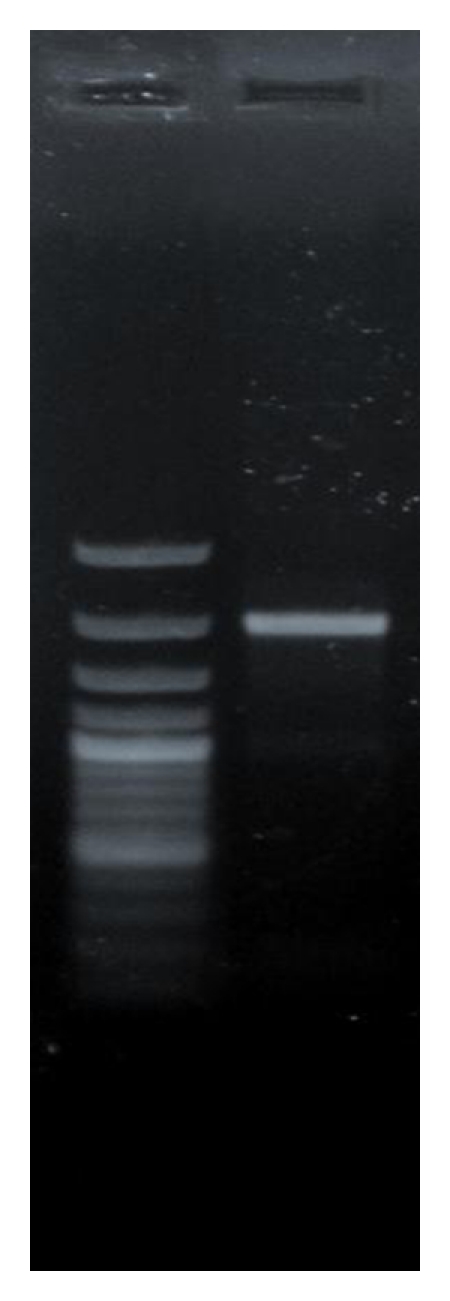
PCR Amplification of HSP70-1 gene. [Lane 1: 100 bp plus Marker, Lane 2: PCR product at 49°C anealing temperature].

The concentration of RNA was checked by analyzing OD260 /OD280 ratio which was found in the range of 1.8-1.9 indicated the purity of the RNA, and the yield was obtained in range of 3.5–3.9 *μ*g/mL. From the obtained total RNA, cDNA was synthesized and PCR amplification was carried out. Agarose gel electrophoresis revealed 1926-bp PCR product of HSP70-1 gene on 1% agarose gel (Figure 1). Restriction enzyme Bsr I was cut at ACTGGN restriction site and yielded two fragments, 1211 bp and 715 bp. The presence of HSP70-1 gene insert in plasmid was further confirmed by PCR. The gene insert (1926 bp) was released from the recombinant pGEM-T easy plasmid using EcoR I. GAATTC is restriction site for EcoR I which releases gene insert from recombinant plasmids. The plasmids were further characterized by Sac I and Pst I. The Sac I cuts vector at position 110 (restriction site- GAGCTC) leading to linearization of recombinant vector resulting in 4926-bp band. Pst I having two restriction sites (CTGCAG) one in vector (89) and other in gene insert (1052) could produce 3963 and 963 bp fragments in case of right orientation or 3785 and 1141 bp fragments in case of left orientation of insert. Upon restriction analysis of selected recombinant plasmid with Pst I, bands of 3963 bp and 963 bp size were observed thus confirming the right orientation of inserted gene in vector. The sequencing of this plasmid revealed that HSP70-1 cDNA of goat has full length ORFs of 1926 bp, as reported for other ruminants like cattle, buffalo [14, 15]. The sequence was submitted to NCBI Genbank, and the accession number: **FJ975769** for goat HSP70-1 gene complete cds was obtained.

### 3.2. Sequence Analysis

**Figure 2:**
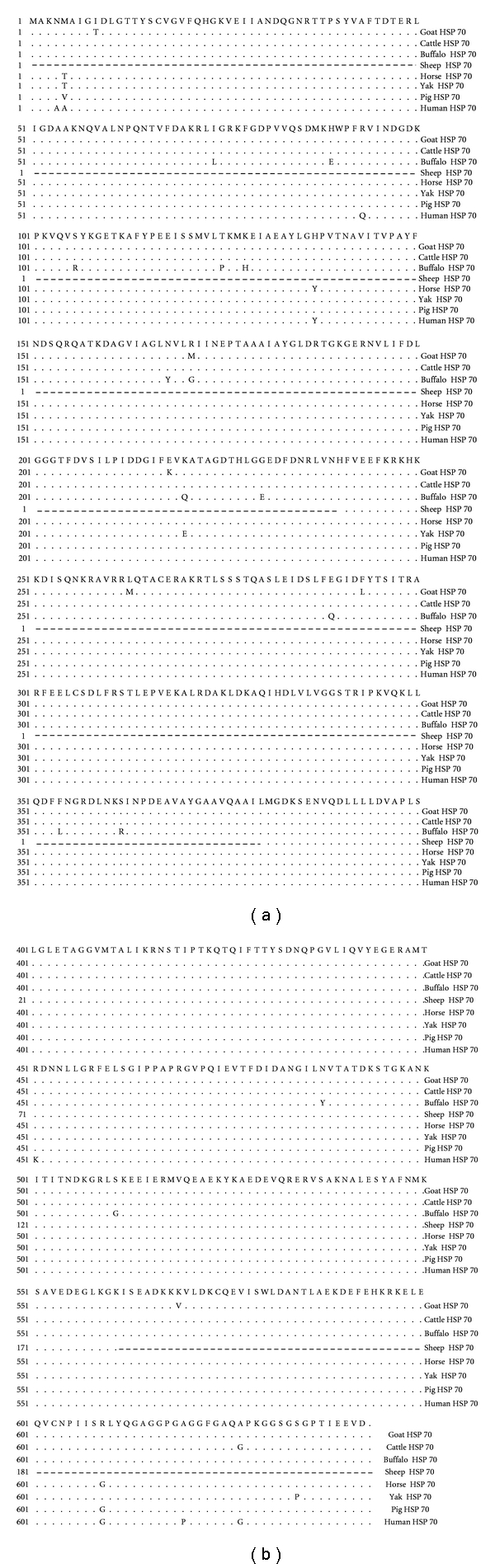
Alignment of predicted amino acid sequence of goat HSP70 with different domestic species and human. Identical sequence is indicated by a dot and differences by the corresponding one-letter symbol of the amino acid. Dark grey boxes indicate highly conserved sequences at positions 9–16, 131–139, and 388–396. Individual grey boxes show substituted amino acids.

The nucleotide sequence and predicted amino acid sequence were aligned and compared with all HSP70-1 cDNA sequences of different domestic, species namely, goat (FJ_975769), cattle (NM_174550), buffalo (EU_099315.1), yak (DQ_022675.1), sheep (partial- AJ_812230.1), pig (AK_235308.1), horse (XM_001492096), and human (AK_301243.1) using Clustal W method of MegAlign module in DNASTAR Version 4.0, Inc., USA which revealed the nucleotide substitutions. Only partial sequence was available in sheep but being a species related to goat, the sheep (partial) sequence was used in phylogenic analysis. The entire nucleotide sequence of goat HSP70-1 gene shows 97.8% homology with cattle, 96.3% with buffalo, 97.5% with yak, 99.4% with sheep (partial), 95.3% with pig, 94.4% with horse, and 94.1% with human which indicates close evolutionary relationship. Madhusudan [15] observed that buffalo HSP70 cDNA sequence and deduced amino acid has 97.8% and 94.4% homology, respectively, with cattle HSP70-1 DNA sequence and deduced amino acid sequence. Among the thirty nine nucleotides substitutions in cDNA sequence of HSP70-1 gene of goat, as compared to cattle, changes at codon positions 26, 512, 652, 787, and 1706 resulted in amino acid substitutions (Figure 2). Inferred amino acid sequence of 641 residues of goat HSP70 gene was 98.6% similar to cattle, 95.9% to buffalo, 98.4% to yak, 100% to sheep (partial), 98% to pig, 98.1% to horse, and 97.7% to human sequence. 

The results indicate that goat HSP70-1 nucleotide and deduced amino acid sequence is highly conserved across the species. Pelham [16] reported that HSP proteins are highly conserved both in protein coding sequence and in regulatory sequence. Gutierrez and Guerriero [14] found that amino acid sequences 9–16 and 131–139 were highly conserved in the HSP70 family of proteins. In goat HSP70, amino acid threonine replaced isoleucine at position 9, and sequence from 10–16 and 131–139 was found to be conserved. Morimoto et al. [17] characterized chicken HSP70 gene and found that chicken HSP70 cDNA sequence and deduced amino acid sequence is 80% identical to human, HSP70 cDNA sequence and deduced amino acid sequence, whereas 73% identical to Drosophila HSP70 cDNA sequence and 71% identical to Drosophila HSP70 amino acid sequence. The amino acid sequence of HSP70 gene in canine showed 90%–95% sequence similarity with bovine, human, and mouse HSP70 proteins [18].

### 3.3. Phylogenetic Analysis

**Figure 3:**
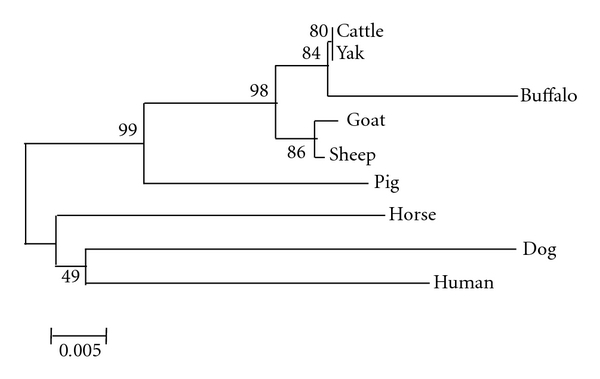
Phylogenetic relationship of the HSP70-1 nucleotide sequences from different species using Mega version 4.1 following the alignment of the ORF sequences using Clustal W and neighbor-joining method (nucleotide p distance). Numbers outside the branches indicate the bootstrap values obtained using 1,000 replicates, and values above 50% are shown. Scale bar at the bottom measures the nucleotide distance.

Based on the nucleic acid sequences of HSP70-1 full length ORF, phylogenetic tree was drawn by Mega 4.0 [19] considering 1,000 bootstrap values (Figure 3). It was found that ruminants and monogastrics are derived from different clusters according to their closer evolutionary relationship. Among ruminants, cattle, buffalo, goat, and sheep might have evolved from a common ancestor; pig positioned in between and diverged early from the bovid ancestors. Selection pressure, as determined by codon-based *Z* test using the Nei Gojobori method, revealed that at 5% level of significance, dN is substantially greater than dS. Thus, HSP70 might have evolved by positive selection (dN > dS) among these species. Goat and sheep HSP70-1 gene showed identical lineage. However, cattle, buffalo, and yak are having similarity with goat having different lineage. Pig, horse, and human sequences show dissimilarities suggesting different ancestry.

### 3.4. Predicted Caprine HSP70 Protein

**Figure 4:**
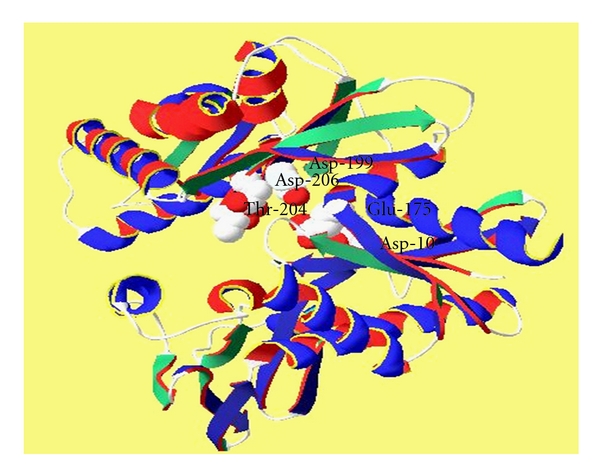
Caprine HSP70 Protein (Amino acids involved in ATPase-Hydrolytic Cycle).

Predicted HSP70 protein of goat possesses molecular weight of 70190.56 Da. with 641 amino acids of which 82 are strongly basic (+) amino acids (K, R) and 92 are strongly acidic (−) amino acids (D, E). Further, 220 amino acids (A, I, L, F, W, V) are hydrophobic and 151 amino acids (N, C, Q, S, T, Y) are polar in nature. Isoelectric point of HSP70 protein in goat is 5.611 and has got charge of −8.829 at pH 7.0. All the HSP70 proteins consist of same working parts; a highly conserved NH_2_-terminal ATPase domain of 44 kDa and a COOH-terminal region of 25 kDa. Further, COOH-terminal region is divided into a conserved substrate binding domain of 15 kDa and a less conserved immediate COOH-terminal domain of 10 kDa [20]. HSP70 chaperones, with the assistance of cochaperones, serve as cellular machinery that performs many protein folding processes in almost all cellular compartments. In stress conditions misfolding and aggregation of proteins is prevented by HSP70 protein through its ATP-regulated association with short hydrophobic segments in substrate polypeptides [21, 22]. Certain amino acid residues possess a role in ATPase hydrolytic cycle. Hydrogen of hydroxyl group of Thr-204 bonds with inorganic phosphate (Pi) when MgADP and Pi are bound to the ATPase fragment. Asp-10 and Asp-199 bind to the divalent Mg^2+^ ion when Mg^2+^ ADP is bound to HSP70 [23]. Asp-10 and Asp-199 both form hydrogen bond to two H_2_O molecules in the first coordination shell of Mg^2+^ whereas Glu-175 appears to form a weak hydrogen bond to one of these H_2_O molecules. Asp-206 has the smallest effect on catalytic activity as compared to Asp-10, Asp-199, and Glu-175 [24]. Glu-175 is at the same position in the three-dimensional structure as an aspartic acid which is proposed to be a catalytic base in hexokinase [25]. Asp-10, Asp-199, Asp-206 and Glu-175 are catalytically essential acidic residues in the active site region of the ATPase fragment of HSP70 which were found conserved in caprine HSP70 protein as well (Figure 4).

HSP70 uncoats clathrin-coated vesicles both *in vivo* [26, 27] and *in vitro* conditions [28–30]. Mutations that affect interdomain communication affect clathrin cage disassembly. I216 and I515 that are involved in extensive interdomain hydrophobic contact along with V519 are important in disassembly of clathrin cages. Similarly, V388 and L393 in the linker sequence also possess this function. E530 or K524 residues involved in ionic interactions do not markedly affect disassembly activity [31]. I216, I515, V519, V388, L393, E530, and K524 are important for uncoating of clathrin-coated vesicles both *in vivo* and *in vitro* and are conserved in goat HSP70 protein. The mutation in I216 shows a large reduction in auxillin binding (20-fold or more), while changes in K325, V388, and L393 exhibit more modest (2.5- to 7-fold) reductions in binding [31]. I216, K325, V388, and L393 which were involved in auxilin binding were found conserved in caprine HSP70 protein. 

Comparison of archaeal, eubacterial, eukaryotic, and organellar HSP70 sequences identifies C-terminal half of the linker (bHSP70 388-396, VQDLLLLDV) as a well-conserved element in the HSP70s [32]. Similar element of linker sequence was found in caprine HSP70 protein.

## 4. Conclusion

Goat Heat shock protein 70-1 cDNA encoding HSP70 protein of 641 amino acid residues was found highly conserved among domestic animals.
